# Total and high molecular weight adiponectin have similar utility for the identification of insulin resistance

**DOI:** 10.1186/1475-2840-9-26

**Published:** 2010-06-23

**Authors:** Paloma Almeda-Valdes, Daniel Cuevas-Ramos, Roopa Mehta, Francisco J Gomez-Perez, Ivette Cruz-Bautista, Olimpia Arellano-Campos, Mariana Navarrete-Lopez, Carlos A Aguilar-Salinas

**Affiliations:** 1 Departamento de Endocrinología y Metabolismo Instituto Nacional de Ciencias Médicas y Nutrición "Salvador Zubirán" 14000 México D.F., México

## Abstract

**Background:**

Insulin resistance (IR) and related metabolic disturbances are characterized by low levels of adiponectin. High molecular weight adiponectin (HMWA) is considered the active form of adiponectin and a better marker of IR than total adiponectin. The objective of this study is to compare the utility of total adiponectin, HMWA and the HMWA/total adiponectin index (S_A _index) for the identification of IR and related metabolic conditions.

**Methods:**

A cross-sectional analysis was performed in a group of ambulatory subjects, aged 20 to 70 years, in Mexico City. Areas under the receiver operator characteristic (ROC) curve for total, HMWA and the S_A _index were plotted for the identification of metabolic disturbances. Sensitivity and specificity, positive and negative predictive values, and accuracy for the identification of IR were calculated.

**Results:**

The study included 101 men and 168 women. The areas under the ROC curve for total and HMWA for the identification of IR (0.664 *vs*. 0.669, *P *= 0.74), obesity (0.592 *vs*. 0.610, *P *= 0.32), hypertriglyceridemia (0.661 *vs*. 0.671, *P *= 0.50) and hypoalphalipoproteinemia (0.624 *vs*. 0.633, *P *= 0.58) were similar. A total adiponectin level of 8.03 μg/ml was associated with a sensitivity of 57.6%, a specificity of 65.9%, a positive predictive value of 50.0%, a negative predictive value of 72.4%, and an accuracy of 62.7% for the diagnosis of IR. The corresponding figures for a HMWA value of 4.25 μg/dl were 59.6%, 67.1%, 51.8%, 73.7% and 64.2%.

The area under the ROC curve of the S_A _index for the identification of IR was 0.622 [95% CI 0.554-0.691], obesity 0.613 [95% CI 0.536-0.689], hypertriglyceridemia 0.616 [95% CI 0.549-0.683], and hypoalphalipoproteinemia 0.606 [95% CI 0.535-0.677].

**Conclusions:**

Total adiponectin, HMWA and the S_A _index had similar utility for the identification of IR and metabolic disturbances.

## Background

Adiponectin is a peptide hormone produced by adipose tissue, involved in glucose and lipid metabolism [1-4]. The high molecular weight form is considered the active fraction of adiponectin; for this reason it is considered a better marker of metabolic disturbances than total adiponectin [5-7].

The average plasma concentration of this hormone ranges between 5 and 10 μg/ml; levels vary according to sex, body fat distribution, and metabolic status [8]. Men have approximately 15% lower levels compared to women [9]. Adiponectin concentration is lower in obesity, specifically central obesity [10] and studies confirm that levels increase with weight loss [11]. Low adiponectin concentrations have also been registered in individuals with coronary artery disease [12]. Finally, persons with diabetes have lower concentrations of this hormone compared to non- diabetics; levels are also low in states of hyperinsulinemia and glucose intolerance. Moreover, when adjusting for weight and body fat percentage, insulin sensitivity is independently associated with the adiponectin concentration [13].

The quantification of insulin resistance is desirable in clinical practice since this metabolic state is a treatable precursor of diabetes mellitus [14]. Established direct methods to quantify insulin sensitivity, such as the hyperinsulinemic euglycemic clamp, are complex and time consuming. Surrogate indexes are available, but there are no universal cutoff points to define IR. Adiponectin may be a useful marker for insulin resistance and a variable that can integrate the abnormalities of the metabolic syndrome. As yet, few studies have determined the utility of adiponectin for this purpose. In addition, it is not know whether the high molecular weight form is superior in the evaluation of IR. The aim of this study is to evaluate the utility of total adiponectin, HMWA and the S_A _index for the identification of IR and metabolic disturbances in a Mexican population.

## Methods

### Participants

Ambulatory subjects aged 20 to 70 yr were recruited between March and July 2008 in Mexico City. The population consisted of medium-income healthy workers without a personal history of metabolic abnormalities. To avoid confounding factors known to affect plasma adiponectin concentration the following exclusion criteria were applied: fever (temperature ≥ 38°C), hospitalization in the previous two weeks, known diabetes, arterial hypertension, dyslipidemia, coronary heart disease or a condition with equivalent risk (carotid disease, peripheral artery disease, and aortic aneurysm), congestive heart failure, stroke, chronic renal disease, active hepatic disease, chronic diseases or any other acute or chronic inflammatory illness. These criteria were evaluated by a questionnaire and in the case of diabetes, renal insufficiency and hypertension by the measurement of fasting glucose, serum creatinine and blood pressure. Subjects were also excluded if they were on any medication known to affect the metabolic profile (anti-hypertensives, statins, fibrates, metformin, thiazolidinediones, and salicylic acid). The Ethics Committee approved the protocol and all participants gave written informed consent.

### Study protocol

A questionnaire was administered to all participants; demographic data and medical history including smoking habit were obtained. Height, weight, waist circumference, and blood pressure were measured. Blood pressure was measured twice, after a 5 minute rest between measurements, and the average value was used for data analysis. Blood samples were collected after a 9 to 12-hour fasting period, centrifugated, and refrigerated at -70°C. All assays were carried out on serum samples (except for adiponectin that was measured in plasma) in the laboratory of the Department of Endocrinology and Metabolism. This laboratory is certified by the External Comparative Evaluation of Laboratories Program of the College of American Pathologists. Glucose was measured using the glucose oxidase method (Boehringer Mannheim, Germany), total cholesterol, and triglycerides were measured using an enzymatic method (Boehringer Mannheim, Germany), HDL-cholesterol was measured after precipitation using phosphotungstic acid and Mg^2+^, low density lipoprotein (LDL)-cholesterol concentration was estimated by the Friedewald formula, plasma insulin was determined by a MEIA assay (Abbott Laboratories), total adiponectin and HMWA concentrations were measured using commercial assays (Millipore, Mexico).

Briefly, the HMWA assay is a sandwich ELISA. Firstly the adiponectin molecule is captured by a monoclonal anti-adiponectin antibody coating the wells; secondly a polyclonal anti-adiponectin antibody binds to the captured molecules. Finally, a streptavidin-horseradish peroxidase conjugate binds to this complex. The enzyme activity is measured spectrophotometrically at 450 nm. Prior treatment of the plasma samples removes hexameric and trimeric adiponectin allowing for the specific measurement of HMWA.

### Definitions

Obesity was defined as a BMI of at least 30 kg/m^2^, hypertriglyceridemia was considered present with a triglyceride level ≥ 150 mg/dl, hypoalphalipoproteinemia was defined as HDL-cholesterol < 40 mg/dl in men and < 50 mg/dl in women. IR was evaluated and considered present when either: 1) the homeostasis model assessment of insulin resistance (HOMA-IR) was ≥ 2.5; 2) insulin sensitivity (HOMA2%S) estimated by the HOMA2 calculator v2.2 (Diabetes Trials Unit, University of Oxford) was < 75%, and 3) the triglyceride/HDL-cholesterol index was ≥ 3. The MS was defined according to the National Education Cholesterol Education Program's Adult Treatment Panel III (ATP-III) and the International Diabetes Federation (IDF) criteria [15,16].

### Statistics

Normally distributed data, identified using Kolmogorov-Smirnov test, were expressed as mean ± standard deviation (SD). Variables with a skewed distribution were reported as median [interquartile range] [IQR]. Differences between groups were analyzed with the U-Mann Whitney or Kruskal-Wallis tests. Spearman's correlation coefficients were used to explore the association between total adiponectin, HMWA and S_A _index and IR estimations. ROC curves were plotted and comparisons of the area under the curve were performed as described by De Long *et al. *[17] using Stata version 10 (College Station, TX). Other statistical analyses were performed using SPSS Software version 15 (SPSS, Inc., Chicago IL.).

## Results

The study included 269 individuals (101 men and 168 women). The mean age was 40.1 ± 9.46 years and the mean BMI was 27.7 ± 4.26 kg/m^2^. The study population characteristics are shown in Table 1. Using the ATP-III definition, 28.6% of the study population fulfilled criteria for the MS. When using the IDF criteria the prevalence of the MS rose to 37.9%.

**Table 1:** Characteristics of the study population

	Total *(n = 269)*	Men *(n = 101)*	Women *(n = 168)*	*P*
**Age (yr)**	40.1 ± 9.49	40.5 ± 10.15	39.9 ± 9.42	0.582
**Systolic pressure (mmHg)**	111.1 ± 15.13	115.2 ± 15.00	108.5 ± 14.30	0.001
**Diastolic pressure (mmHg)**	74.5 ± 11.55	77.7 ± 9.98	72.6 ± 11.84	< 0.001
**BMI (kg/m^2^)**	27.7 ± 4.26	27.7 ± 3.81	27.7 ± 4.72	0.465
**Glucose (mmol/liter)**	4.92 ± 1.00	4.93 ± 0.58	4.91 ± 1.30	0.088
**Triglycerides (mmol/liter)**	1.46 [0.77]	1.61 [0.94]	1.37 [0.81]	< 0.001
**Cholesterol (mmol/liter)**	5.20 [1.42]	5.12 [1.61]	5.21 [1.44]	0.987
**HDL-c (mmol/liter)**	1.16 [0.36]	1.01 [0.28]	1.21 [0.44]	< 0.001
**LDL-c (mmol/liter)**	3.34 [1.08]	3.36 [1.20]	3.32 [1.05]	0.850
**Insulin (pmol/liter)**	65.28 [40.97]	68.75 [45.83]	63.19 [39.44]	0.180

Values are means ± SD, or median [IQR]. Number of subjects in each group is indicated in parentheses.

### Plasma total adiponectin and HMWA levels in the study population

There was a close correlation between the concentration of total adiponectin and HMWA (r = 0.890, *P *< 0.001). There was also a moderate concordance between individuals with total and HMWA below and above percentile 50 (kappa = 0.732, *P *< 0.001).

Consistent with the results of previous studies [7,9], total adiponectin and HMWA levels were higher in women than in men (total adiponectin 9.49 [5.31] *vs*. 6.85 [2.78] μg/ml, *P *< 0.001, HMWA 5.7 [4.25] *vs*. 3.5 [2.65] μg/ml, *P *< 0.001). Thirty two participants (22.8% of women) were post-menopausal. Adiponectin and HMWA concentrations did not differ between pre and post-menopausal women (total adiponectin 9.5 [4.72] *vs*. 9.9 [6.48] μg/ml, *P *= 0.122, HMWA 5.7 [3.90] *vs*. 6.5 [6.10] μg/ml, *P *= 0.067). Total adiponectin and HMWA concentrations were lower in obese *vs*. non-obese individuals (total adiponectin 7.56 [3.63] *vs*. 8.7 [4.40] μg/ml, *P *= 0.028, HMWA 4.10 [2.82] *vs*. 5.00 [3.90] μg/ml, *P *= 0.008).

In addition, when comparing subjects with or without the MS the concentrations of total and HMWA were significantly lower in subjects who fulfilled MS criteria. When classifying the participants by gender, this difference was not significant for men (Table 2).

**Table 2:** Concentrations of total and HMWA in patients with and without the metabolic syndrome

	Total adiponectin (μg/ml)	*P*	HMWA (μg/ml)	*P*
**ATP criteria**				
**All *(n = 269)***				
**Metabolic syndrome *(n = 77)***	7.26 [3.57]	0.006	4.10 [3.07]	0.007
**No metabolic syndrome *(n = 192)***	8.91 [4.84]		5.05 [4.03]	
**Men *(n = 101)***				
**Metabolic syndrome *(n = 30)***	6.44 [2.94]	0.183	3.25 [2.13]	0.245
**No metabolic syndrome *(n = 71)***	7.32 [2.65]		3.50 [3.00]	
**Women *(n = 168)***				
**Metabolic syndrome *(n = 47)***	8.68 [4.58]	0.024	4.60 [5.00]	0.037
**No metabolic syndrome *(n = 121)***	10.06 [4.91]		5.80 [4.20]	
**IDF criteria**				
**All *(n = 269)***				
**Metabolic syndrome *(n = 102)***	7.27 [3.39]	< 0.001	3.95 [2.48]	< 0.001
**No metabolic syndrome *(n = 167)***	9.11 [4.98]		5.40 [4.40]	
**Men *(n = 101)***				
**Metabolic syndrome *(n = 45)***	6.68 [2.79]	0.123	3.20 [1.80]	0.114
**No metabolic syndrome *(n = 56)***	7.52 [2.93]		3.55 [3.48]	
**Women *(n = 168)***				
**Metabolic syndrome *(n = 57)***	8.68 [4.33]	0.002	4.60 [4.55]	0.007
**No metabolic syndrome *(n = 111)***	10.48 [4.71]		6.20 [4.40]	

Values are medians [IQR]. Number of subjects in each group is indicated in parentheses.

### Concentration of total and HMWA levels associated with insulin resistance

**Figure 1:**
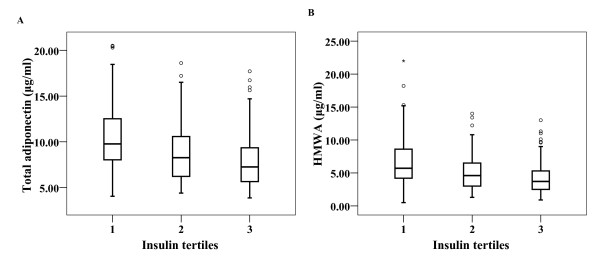
**Box plots of the concentrations of total and HMWA divided by insulin tertiles**. A. Change in total adiponectin levels by insulin tertiles (9.77 [4.58] *vs*. 8.27 [4.42] *vs*. 7.15 [3.79] μg/ml, *P *< 0.001). B. Change in HMWA levels by insulin tertiles (5.7 [4.45] *vs*. 4.6 [3.6] *vs*. 3.7 [2.85] μg/ml, *P *< 0.001).

We evaluated total and HMWA concentrations by insulin tertiles. Both forms of adiponectin decreased significantly across the three insulin tertiles (total adiponectin 9.77 [4.58] *vs*. 8.27 [4.42] *vs*. 7.15 [3.79] μg/ml, *P *< 0.001), HMWA 5.7 [4.45] *vs*. 4.6 [3.6] *vs*. 3.7 [2.85] μg/ml, *P *< 0.001) (Figure 1).

A significant correlation was found between total adiponectin and the surrogate markers for insulin resistance. An almost identical correlation was encountered between HMWA and these markers; however, the correlation was lower for the S_A _index (Table 3).

**Table 3:** Correlation between total adiponectin, HMWA and S_A _index and estimators of IR

	Adiponectin		High Molecular Weight Adiponectin		HMWA/adiponectin	
	
	ρ	*P*	ρ	*P*	ρ	*P*
**Fasting insulin**	-0.330	0.001	-0.323	0.001	-0.230	0.001
**HOMA-IR**	-0.332	0.001	-0.325	0.040	-0.228	0.001
**HOMA2S**	-0.329	0.001	-0.323	0.001	-0.231	0.001
**Triglyceride/HDL cholesterol**	-0.458	0.001	-0.459	0.001	-0.302	0.001

HOMA-IR: homeostasis model assessment of insulin resistance

HOMA2S: insulin sensitivity estimated by the HOMA2 calculator v2.2 (Diabetes Trials Unit, University of Oxford)

Total and HMWA concentration were significantly lower in individuals with IR estimated using HOMA-IR, triglyceride/HDL-cholesterol index and HOMA2%S. When dividing the population by gender this difference remained significant for all the surrogates of IR except HOMA2%S in males (Table 4).

**Table 4:** Concentrations of total and HMWA in patients with and without insulin resistance

	Total adiponectin (μg/ml)	*P*	HMWA (μg/ml)	*P*
**HOMA-IR**				
**All *(n = 269)***				
**Insulin resistance *(n = 100)***	7.05 [3.70]	< 0.001	3.70 [2.90]	< 0.001
**No insulin resistance *(n = 169)***	8.96 [4.78]		5.30 [4.50]	
**Men *(n = 101)***				
**Insulin resistance *(n = 42)***	6.20 [3.15]	0.049	3.15 [2.43]	0.046
**No insulin resistance *(n = 59)***	7.34 [2.65]		3.80 [2.80]	
**Women *(n = 168)***				
**Insulin resistance *(n = 58)***	8.73 [3.66]	< 0.001	4.70 [3.35]	< 0.001
**No insulin resistance *(n = 110)***	10.76 [5.36]		6.45 [4.58]	
				
**Triglyceride/HDL-cholesterol**				
**All *(n = 269)***	7.33 [3.51]	< 0.001	3.90 [3.08]	< 0.001
**Insulin resistance *(n = 156)***	9.90 [4.84]		5.90 [4.20]	
**No insulin resistance *(n = 113)***				
**Men *(n = 101)***	6.70 [3.03]	0.024	3.20 [2.15]	0.004
**Insulin resistance *(n = 78)***	8.17 [2.98]		5.10 [4.40]	
**No insulin resistance *(n = 23)***				
**Women *(n = 168)***	8.70 [4.44]	< 0.001	4.75 [4.45]	< 0.001
**Insulin resistance *(n = 78)***	10.85 [4.73]		6.40 [4.43]	
**No insulin resistance *(n = 90)***				
**HOMA2%S**				
**All *(n = 269)***				
**Insulin resistance *(n = 123)***	7.97 [3.74]	< 0.001	4.20 [3.23]	< 0.001
**No insulin resistance *(n = 146)***	8.70 [4.74]		4.95 [4.58]	
**Men *(n = 101)***				
**Insulin resistance *(n = 47)***	6.61 [3.28]	0.115	3.20 [2.80]	0.240
**No insulin resistance *(n = 54)***	7.33 [2.71]		3.55 [2.63]	
**Women *(n = 168)***				
**Insulin resistance *(n = 76)***	9.01 [4.62]	0.001	4.90 [3.70]	<0.001
**No insulin resistance *(n = 92)***	10.76 [5.15]		6.55 [4.63]	

Values are medians [IQR]. Number of subjects in each group is indicated in parentheses.

### Utility of total adiponectin and HMWA for the identification of IR and metabolic disturbances

**Figure 2:**
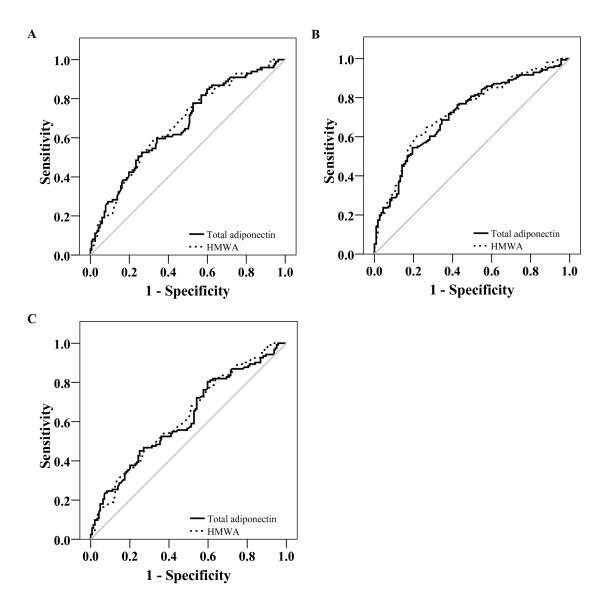
**Area under the ROC curve of total and HMWA for the diagnosis of IR**. A. Area under the ROC curve of total (0.664 [95% CI 0.597-0.732]) and HMWA (0.669 [95% CI 0.603-0.735]) for the identification of IR considering HOMA-IR. B. Area under ROC curve of total (0.717 [95% CI 0.655-0.778]) and HMWA (0.730 [95% CI 0.670-0.790]) for the identification of IR considering triglyceride/HDL-cholesterol index. C. Area under ROC curve of total (0.617 [95% CI 0.549-0.684] and HMWA (0.623 [95% CI 0.556-0.690]) for the identification of IR considering HOMA2%S.

We plotted ROC curves to compare the utility of plasma total adiponectin level, HMWA and S_A _index for the identification of IR and metabolic disturbances. The AUC for total adiponectin was not significantly different to that of HMWA for the identification of IR estimated by HOMA-IR (total adiponectin 0.664 [95% CI 0.597-0.732] *vs*. HMWA 0.669 [95% CI 0.603-0.735], *P *= 0.743), triglyceride/HDL-cholesterol index (total adiponectin 0.717 [95% CI 0.655-0.778] *vs*. HMWA 0.730 [95% CI 0.670-0.790], *P *= 0.417), and HOMA2%S (total adiponectin 0.617 [95% CI 0.549-0.684] *vs*. HMWA 0.623 [95% CI 0.556-0.690], *P *= 0.698) (Figure 2).

In addition, the area under the ROC curves for total adiponectin and HMWA were not significantly different for the identification of metabolic abnormalities; obesity (total adiponectin 0.592 [95% CI 0.510-0.673] *vs*. HMWA 0.610 [95% CI 0.531-0.690], *P *= 0.32); hypertriglyceridemia (total adiponectin 0.661 [95% CI 0.596-0.726] *vs*. HMWA 0.671 [95% CI 0.607-0.736], *P *= 0.504); and hypoalphalipoproteinemia (total adiponectin 0.624 [95% CI 0.555-0.692] *vs*. HMWA 0.633 [95% CI 0.564-0.702], *P *= 0.582).

The utility of the S_A _index for the identification of IR and related metabolic disturbances showed an inferior performance compared with total adiponectin and HMWA concentrations; for IR estimated by HOMA 0.622 [95% CI 0.554-0.691], triglyceride/HDL-cholesterol index 0.663 [95% CI 0.598-0.729], and HOMA2%S 0.603 [95% CI 0.535-0.671]. The areas under the ROC curves were also lower for the identification of metabolic abnormalities; obesity 0.613 [95% CI 0.536-0.689], hypertriglyceridemia 0.616 [95% CI 0.549-0.683], and hypoalphalipoproteinemia 0.606 [95% CI 0.535-0.677]. When we analyzed the population stratifying by gender, the resultant conclusions with respect to the areas under the ROC curves of adiponectin, HMWA and the S_A _index were similar as those derived from the total population (Table 5).

**Table 5:** AUC of total and HMWA for the identification of IR and related metabolic abnormalities

	All *(n = 269)*	Women *(n = 168)*	Men *(n = 101)*
**IR by HOMA-IR**			
**Total adiponectin**	0.664 [0.597-0.732]	0.705 [0.622-0.788]	0.615 [0.501-0.729]
**HMWA**	0.669 [0.603-0.735]	0.693 [0.608-0.777]	0.617 [0.506-0.728]
**S_A _index**	0.622 [0.554-0.691]	0.631 [0.541-0.721]	0.584 [0.472-0.697]
**IR by triglyceride/HDL-cholesterol index**			
**Total adiponectin**	0.717 [0.655-0.778]	0.670 [0.587-0.753]	0.655 [0.530-0.779]
**HMWA**	0.730 [0.670-0.790]	0.661 [0.579-0.744]	0.696 [0.557-0.835]
**S_A _index**	0.663 [0.598-0.729]	0.604 [0.518-0.690]	0.592 [0.475-0.777]
**IR by HOMA2%S**			
**Total adiponectin**	0.617 [0.549-0.684]	0.654 [0.569-0.738]	0.591 [0.479-0.704]
**HMWA**	0.623 [0.556-0.690]	0.664 [0.582-0.747]	0.568 [0.456-0.680]
**S_A _index**	0.603 [0.535-0.671]	0.657 [0.574-0.740]	0.524 [0.411-0.638]
**Obesity**			
**Total adiponectin**	0.592 [0.510-0.673]	0.591 [0.485-0.697]	0.633 [0.507-0.758]
**HMWA**	0.610 [0.531-0.690]	0.608 [0.504-0.713]	0.637 [0.519-0.756]
**S_A _index**	0.613 [0.536-0.689]	0.607 [0.510-0.705]	0.637 [0.518-0.756]
**Hypertriglyceridemia**			
**Total adiponectin**	0.661 [0.596-0.726]	0.650 [0.561-0.738]	0.550 [0.434-0.666]
**HMWA**	0.671 [0.607-0.736]	0.634 [0.545-0.724]	0.604 [0.488-0.720]
**S_A _index**	0.616 [0.549-0.683]	0.572 [0.480-0.664]	0.586 [0.471-0.701]
**Hypoalphalipoproteinemia**			
**Total adiponectin**	0.624 [0.555-0.692]	0.621 [0.535-0.707]	0.672 [0.565-0. 780]
**HMWA**	0.633 [0.564-0.702]	0.631 [0.545-0.716]	0.657 [0.538-0.776]
**S_A _index**	0.606 [0.535-0.677]	0.604 [0.516-0.691]	0.597 [0.473-0.721]

Values are areas [95% confidence interval]. Number of subjects in each group is indicated in parentheses.

A total adiponectin level of 8.03 μg/ml was associated with a sensitivity of 57.6%, a specificity of 65.9%, a positive predictive value of 50.0%, a negative predictive value of 72.4%, and an accuracy of 62.7% for the diagnosis of IR using the HOMA-IR index. The corresponding figures for a HMWA level of 4.25 μg/dl were 59.6%, 67.1%, 51.8%, 73.7% and 64.2%, respectively. When using the triglyceride/HDL-cholesterol index, the sensitivity of a total adiponectin level of 7.76 μg/ml was 54.5%, specificity 80.5%, positive predictive value 79.4%, negative predictive value 56.2%, and accuracy 65.4%. The corresponding figures for a HMWA level of 4.65 μg/ml were 64.7%, 73.5%, 77.1%, 60.1%, and 68.4%, respectively. Finally, when considering the HOMA2%S value, a total adiponectin value of 8.25 μg/ml yielded a sensitivity of 52.4%, specificity 59.9%, positive predictive value 53.3%, negative predictive value 59%, and accuracy of 56.3%. The corresponding figures for a HMWA level of 4.25 μg/ml were 52%, 66.3%, 75.4%, 41.0%, and 56.7%.

## Discussion

In recent years, several markers have been proposed for the screening, diagnosis, and therapeutic monitoring of insulin resistant patients [18]. However, all have problems that limit their use to research studies [19]. Not one succeeds in integrating the global assessment of the metabolic abnormalities that may increase the risk for developing type 2 diabetes and cardiovascular outcomes. Adiponectin is a promising biomarker of insulin resistance. Low adiponectin levels are associated with the majority of the metabolic syndrome traits and are related to an increased risk for having type 2 diabetes [20,21]. This hormone is not prone to degradation and is only minimally affected by diurnal variations, pre- vs. postprandial status, and acute infections. Adiponectin levels can be assessed by either of three variables: total adiponectin, HMWA and the S_A _index. Here, we compare the diagnostic properties of these variables for detecting insulin resistance (assessed using surrogate markers) and other metabolic abnormalities in a population composed of apparently healthy adults. Total adiponectin, HMWA and the S_A _index had similar utility for the identification of the metabolic abnormalities. Our results suggest that plasma total adiponectin may provide clinical information of the same diagnostic value as HMWA. This finding may stimulate the use of adiponectin in clinical and epidemiological settings because the measurement of total adiponectin is better standardized, cheaper and more accessible than the other two approaches.

The highly significant correlation with insulin resistance makes adiponectin a powerful marker for diabetic risk. Several groups have shown that a low adiponectin level predicts the incidence of diabetes in children [22] and adults from several ethnic backgrounds [23]. Its high molecular weight form is the active fraction of adiponectin, is selectively down-regulated in IR states [5,14], and has been proposed as the best approach to evaluate adiponectin concentrations. However, the studies that support this conclusion have a small sample size [24], are *post hoc *analysis of studies designed for other purposes [25] or include biased populations (i.e. individuals with cardiovascular disease) [5]. For example, Basu *et al. *evaluated adiponectin complex distribution in 11 type 2 diabetic and 7 nondiabetic subjects. Total adiponectin was lower in the diabetic subjects primarily due to a reduction of HMWA [24]. However, the limited sample size of this study does not allow for the evaluation of adiponectin or HMWA as a diagnostic test for IR. Hara *et al. *reported that HMWA was better than total adiponectin for the detection of IR [5]. In this study, the population consisted of patients with diabetes or those who had had a coronary angiography. The authors estimated IR (by calculating the HOMA index) and they reported a greater area under the ROC curve for HMWA of 0.713 (95% CI 0.620-0.805) than that found for total adiponectin of 0.615 (95% CI 0.522-0.708, *P *= 0.016). In our study we investigated the utility of total *vs*. HMWA for the identification of IR and related metabolic disturbances in an adult Mexican cohort. This population had no factors known to interfere with adiponectin concentrations. Our results showed a similar area under the ROC curve for both total adiponectin 0.664 [95% CI 0.597-0.732] and HMWA 0.669 [95% CI 0.603-0.735], *P *= 0.743). The same conclusion was reached for all surrogate markers of insulin resistance. We believe that our results differ from previous reports because of the characteristics of the population under study. Our study sample is representative of persons in whom a clinical indicator of IR is needed for early identification of diabetes risk.

In addition, no difference between the three approaches was found in the areas under the ROC curves for the identification of other metabolic disturbances (obesity, hypertriglyceridemia, hypoalphalipoproteinemia and IR). These findings suggest that adiponectin is of value for the identification of IR and metabolic disturbances. If this turns out to be the case, adiponectin may be a marker for global metabolic status. For this reason we attempted to estimate an adiponectin threshold for the identification of IR. The cutoff points used had a reasonable sensitivity and specificity. Further studies are needed to assess the diagnostic performance of these cutoff points in other populations.

One of the disadvantages of adiponectin is that the assay for its measurement is not widely available and is expensive. In addition, the HMWA is even more expensive and difficult to implement compared to the total adiponectin method. However, adiponectin measurements may be useful in the future to identify patients at risk for metabolic alterations and to monitor treatment results.

The cross sectional design, the use of surrogate markers (instead of the euglucemic hyperinsulinemic clamp) to measure insulin action and a moderate sample size are the main limitations of our study.

## Conclusions

In this study, total adiponectin was as useful as HMWA and the S_A _index in evaluating metabolic status. In addition, there was a similar association between total and HMWA in assessing IR and related metabolic abnormalities.

## Competing interests

The authors declare that they have no competing interests.

## Authors' contributions

PA-V, DC-R and CA-S participated in the design, interpretation of the study, analysis of the data, and review of the manuscript. RM and FG-P participated in the review of the manuscript. IC-B, OA-C and MN-L participated in the methodology of the study. All authors have read and approved the final manuscript.
